# Effect of some operational parameters on the arsenic removal by electrocoagulation using iron electrodes

**DOI:** 10.1186/2052-336X-12-95

**Published:** 2014-06-11

**Authors:** Berrin Zeliha Can, Recep Boncukcuoglu, Alper Erdem Yilmaz, Baybars Ali Fil

**Affiliations:** 1 Eighth Regional Directorate of State Hydraulic Works (DSİ), Erzurum, Turkey; 2 Department of Environmental Engineering, Engineering Faculty, Atatürk University, Erzurum, Turkey; 3 Department of Environmental Engineering, Faculty of Engineering, Balıkesir University, Balikesir, Turkey

**Keywords:** Arsenic removal, Electrocoagulation, Wastewater treatment, Iron electrode

## Abstract

Arsenic contamination of drinking water is a global problem that will likely become more apparent in future years as scientists and engineers measure the true extent of the problem. Arsenic poisoning is preventable though as there are several methods for easily removing even trace amounts of arsenic from drinking water. In the present study, electrocoagulation was evaluated as a treatment technology for arsenic removal from aqueous solutions. The effects of parameters such as initial pH, current density, initial concentration, supporting electrolyte type and stirring speed on removal efficiency were investigated. It has been observed that initial pH was highly effective on the arsenic removal efficiency. The highest removal efficiency was observed at initial pH = 4. The obtained experimental results showed that the efficiency of arsenic removal increased with increasing current density and decreased with increasing arsenic concentration in the solution. Supporting electrolyte had not significant effects on removal, adding supporting electrolyte decreased energy consumption. The effect of stirring speed on removal efficiency was investigated and the best removal efficiency was at the 150 rpm. Under the optimum conditions of initial pH 4, current density of 0.54 mA/cm^2^, stirring speed of 150 rpm, electrolysis time of 30 minutes, removal was obtained as 99.50%. Energy consumption in the above conditions was calculated as 0.33 kWh/m^3^. Electrocoagulation with iron electrodes was able to bring down 50 mg/L arsenic concentration to less than 10 μg/L at the end of electrolysis time of 45 minutes with low electrical energy consumption as 0.52 kWh/m^3^.

## Introduction

Arsenic, a toxic trace element present in natural waters (ground and surface water), has become a major unavoidable threat for the life of human beings and useful microorganisms. Arsenic concentration in water can become elevated due to several reasons like, mineral dissolution, use of arsenical pesticides, disposal of fly ash, mine drainage and geothermal discharge [1]. Arsenic can exist in four different oxidation states: (-III), (0), (III), and (V), however, oxidized arsenite (As(III)) and arsenate (As(V)) are the most widespread forms in soils and natural waters [2]. Under low pH and mildly reducing conditions (>100 mV), As(III) is thermodynamically stable and exists as arsenious acid (H_3_AsO_3_, H_2_AsO_3_^−^, HAsO_3_^2−^ and AsO_3_^3−^). Under oxidizing conditions, the predominant species is As(V) which exists as arsenic acid (H_3_AsO_4_, H_2_AsO_4_^−^, HAsO_4_^2−^ and AsO_4_^3−^) [2]. As(III) is more mobile in groundwater and 25–60 times more toxic than As(V). The concentration of arsenic species is mainly dependent on redox potentials and pH [3].

Arsenic contamination in potable water supplies is a serious health problem in many countries around the world. As it causes to skin, liver, lung and kidney or bladder cancer, it is a big headache to the nations [4]. Due to carcinogenic nature of arsenic compounds, the purpose should now be to reduce the concentration of arsenic-contaminated water to a level as close to zero as possible. By the World Health Organization, the provisional guideline value for arsenic in drinking water is given as 10 μg/L as a provisional guideline value [5]. Therefore, the drinking water containing arsenic should be treated before usage. Several methods have been investigated for removal of arsenic, including ion exchange [6,7], coagulation and precipitation with iron and aluminum salts [8], adsorption [9-11], electrocoagulation [12-15], membrane techniques like ultrafiltration [16,17], nanofiltration [18], electrodialysis [19], reverse osmosis [13,20,21]. Other techniques like solvent extraction [22], bioremediation [23,24] have been developed for the removal of arsenic too.

In recent years, new processes for efficient and adequate treatment of various industrial wastewaters with relatively low operating costs have been needed due to strict environmental regulations. At this point, the electrocoagulation process has attracted a great deal of attention in treating industrial wastewaters because of its versatility and environmental compatibility [25,26]. Electrocoagulation consists of an in situ generation of coagulants by an electrical dissolution of iron or aluminum electrodes. The metal ions generation takes place at the anode; hydrogen gas is released from the cathode. The hydrogen gas would also help to float the flocculated particles out of the water and therefore the process sometimes is named as electroflocculation [27]. Typically, aluminum, iron, carbon, mild steel, graphite and titanium plates are used as electrodes in the electrocoagulation process. But iron and aluminum have been reported to be very effective and successful in pollutant removal at favorable operating conditions. In the iron electrode two mechanisms have been proposed for the production of iron hydroxide, Fe(OH)_n_where n = 2 or 3 [28]:

•Mechanism 1.

 Anode:

(1) $$4Fe_{\left(s\right)}\to 4F{e^{2+}}_{\left(\mathrm{aq}\right)}+8e^{-}$$

(2) $$4F{e^{2+}}_{\left(\mathrm{aq}\right)}+10H_{2}O_{\left(l\right)}+{O_{2}}_{\left(g\right)}\to 4\mathrm{Fe}{{\left(\mathrm{OH}\right)}_{3}}_{\left(s\right)}+8{H^{+}}_{\left(\mathrm{aq}\right)}$$

 Cathode:

(3) $$8{H^{+}}_{\left(\mathrm{aq}\right)}+8e^{-}\to 4{H_{2}}_{\left(g\right)}$$

 Overall:

(4) $$4Fe_{\left(s\right)}+10H_{2}O_{\left(l\right)}+{O_{2}}_{\left(g\right)}\to 4\mathrm{Fe}{{\left(\mathrm{OH}\right)}_{3}}_{\left(s\right)}+4{H_{2}}_{\left(g\right)}$$

•Mechanism 2.

 Anode:

(5) $$Fe_{\left(s\right)}\to F{e^{2+}}_{\left(\mathrm{aq}\right)}+2e^{-}$$

(6) $$F{e^{2+}}_{\left(\mathrm{aq}\right)}+2O{H^{-}}_{\left(\mathrm{aq}\right)}\to \mathrm{Fe}{{\left(\mathrm{OH}\right)}_{2}}_{\left(s\right)}$$

 Cathode:

(7) $$2H_{2}O_{\left(l\right)}+2e^{-}\to {H_{2}}_{\left(g\right)}+2O{H^{-}}_{\left(\mathrm{aq}\right)}$$

 Overall:

(8) $$Fe_{\left(s\right)}+2H_{2}O_{\left(l\right)}\to \mathrm{Fe}{{\left(\mathrm{OH}\right)}_{2}}_{\left(s\right)}+{H_{2}}_{\left(g\right)}$$

Electrocoagulation is an emerging water treatment technology and could be a good choice to remove As (III) from water: the amount of required chemicals is much lower, a smaller amount of sludge is produced, no mixing of chemical is required, coagulant dosing as well required over potentials can be easily calculated and controlled, operating costs are much lower when compared with most of the conventional Technologies [29,30]. It is felt that As(III) might be oxidized to As(V) during electrocoagulation and gets adsorbed on to the metal hydroxides generated. Electrocoagulation has been successfully used to treat arsenic waste waters, with removal efficiencies as high as 90–99% [1,13,14,31]. It was found that the rate of removal depends on the different operational parameters including initial concentration of arsenic, current density, the influence of pH and electrolysis time. Also groundwater could be cleaned for arsenic by electrochemical generated iron cations by Parga et al. [14]. Hansen et al. found analyzing preliminarily the electrocoagulation process in a modified flow sedimentation basin that it could be obtained a removal of 98% from a 100 ppm As solution [15]. Laboratory scale experiments to remove arsenic by the electrocoagulation process were conducted with three types of electrodes, namely iron, aluminum, and titanium [12]. The highest removal of arsenic (99%) was obtained by using iron electrodes at a pH range of 6–8. It may be because of high adsorption capacity of hydrous ferric oxides for arsenic removal. It was noted that As (III) removal mechanism in EC process seems to be oxidation of As (III) to As (V) and subsequence adsorption on to hydrous ferric oxides. As (III) oxidation to As(V) has previously been proposed to occur with dissolved oxygen and soluble intermediates in Fe(II) oxidation acting as rate enhancing species. As (III) oxidation can also occur when Fe(II) is present with Fe(III) oxyhydroxides, and the mechanism has been proposed to involve the formation of reactive Fe intermediate species.

The results on the investigation of the electrocoagulation process for treatment of arsenic indicated that using an electrocoagulation reactor successfully removes arsenic from water or wastewater. Therefore, the purpose of the present study was to investigate the effect of various operational parameters such as initial pH(2, 3, 4, 5, 6, 7 and 8), electrolysis time (0, 3, 5, 10, 20, 30, 45 and 60 minute), current density (0.18 mA/cm^2^, 0.36 mA/cm^2^, 0.54 mA/cm^2^, 0.71 mA/cm^2^, 0.89 mA/cm^2^ and 1.07 mA/cm^2^), initial arsenic concentration (10 mg/L As, 25 mg/L As, 50 mg/L As and 100 mg/L As), supporting electrolyte type (Na_2_SO_4_, NaCI and KCI) and stirring speed (50 rpm, 150 rpm, 250 rpm and 350 rpm) on the arsenic removal using electrocoagulation method.

## Materials and methods

### Materials

All chemicals were of analytical grade and supplied by Merck and Panreac. Stock arsenic solutions of 1.3 g/L were prepared by dissolving arsenic oxide (As_2_O_3_) in 2N NaOH and then diluted the solution up to 1 liter with de-ionized water. Solutions of lower concentrations were prepared by proper dilution. The pH of the solution was adjusted by adding either concentrated NaOH or H_2_SO_4_.

### Analytical methods

The concentration of As was determined by an atomic absorption spectrophotometer (AAS) model Shimadzu AA 6800 equipped with a hydride generation. Hydride generation is, perhaps, the most popular sample derivatization method used for inorganic arsenic detection, since Holak first reported it in 1969 [32]. Initially it was developed as a method for AAS, whereby sodium or potassium tetrahydroborate (III) is used for arsine production (Eq. 9, 10). The reduction reagents NaBH_4_ and KBH_4_ have proved to be exceptionally reliable reagents for the conversion of the sample to volatile forms [33]. The hydride generation procedure can be also used for differential determination of As (III) and As (V), based on the fact that As (III) reacts with tetrahydroborate at a higher pH than As (V). Thus tetrahydroborate is acting as a reductant for As (V) as well as a hydride source. The inclusion of on-line hydride generation generally increases the sensitivity of detection and reduces the possible interferences from the sample matrix. In this study sodium tetrahydroborate (NaBH_4_) was of analytical grade (Merck) and was dissolved in sodium hydroxide solution just before use.

(9) $$\mathrm{As}{\left(\mathrm{OH}\right)}_{3}+3B{H_{4}}^{-}+3H^{+}\to \mathrm{As}H_{3}+3BH_{3}+3H_{2}O$$

(10) $$BH_{3}+3H_{2}O\to H_{3}BO_{3}+3H_{2}$$

The removal efficiency of As in solution treated by electrocoagulation is calculated as follows:

(11) $$\eta \left(\%\right)=\frac{C_{0}-C_{t}}{C_{0}}x100$$

where, *η* is arsenic removal efficiency, *C*_*0*_, and *C*_*t*_ are the initial arsenic concentration and concentration of arsenic at time *t* in solution (mg/L), respectively. The energy consumption was calculated by the following equation [34];

(12) $$E\left(\mathrm{kWh}/m^{3}\right)=\frac{\mathrm{IxVxt}}{\nu }$$

where, *E* is electrical energy consumption (kWh/m^3^), *V* is potential (volt), *I* is current (ampere), *t* is electrolysis time (min) and *v* is volume of the solution (m^3^). The relative standart deviation during arsenic analysis was in the range of 0-2%.

### Electrocoagulation test

**Figure 1:**
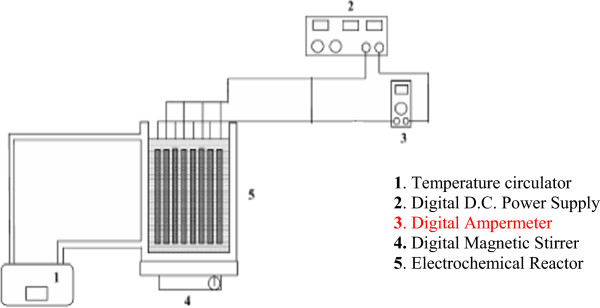
Schematic diagram of the experimental setup.

The experiments carried out in a 1400 mL laboratory-scale batch reactor made of plexiglass. Two groups of alternating electrodes being cathodes and anodes (by six plates of each type) made of iron with total area of approximately 1400 cm^2^ were arranged vertically. The net spacing between the iron electrodes was 0.5 cm. They were treated with the solution of HNO_3_ for cleaning prior to use. At the end of run, the electrodes were washed thoroughly with water to remove any solid residues on the surfaces, and dried. Electrodes were connected to a digital DC power supply characterized by the ranges 0-12 A for current and 0–30V for voltage in monopolar mode. Cell current was measured using Brymen BM–810 multimeter. During the experiments, the electrocoagulation unit was stirred at predetermined speed by a magnetic stirrer (Heidolph MR-3004). The pH and conductivity were measured by a multimeter (WTW, Multiline 340i), which was freshly calibrated by 2 points (4.01; 7.00) before each test. The experimental apparatus is given in Figure 1.

The reactor was fed with 1400 mL of arsenic containing solution at the beginning of each run. The experiment was started by switching the DC power supply on, and then the residual arsenic concentration in samples taken and filtered at predetermined time intervals was measured. The samples were analyzed by atomic absorption spectroscopy (Shimadzu AA 6800) with a hydride generation. In electrocoagulation studies, initial pH, electrolysis time, current density, initial arsenic concentration, supporting electrolyte type and stirring speed were used as parameters whose values are given in Table 1.

**Table 1:** Experimental parameters

**Parameters**	**Chosen parameter ranges**	**Constant variables**
**Initial pH**	2, 3, 4, 5, 6, 7, 8	initial arsenic concentration: 50 mg/L, current density: 0.54 mA/cm^2^, stirring speed: 150 rpm
**Current density, mA/cm**^**2**^	0.18, 0.36, 0.54, 0.71, 0.89, 1.07	initial pH:4, initial arsenic concentration: 50 mg/L, stirring speed: 150 rpm
**Arsenic concentration, mg/L**	10, 25, 50, 100	initial pH:4, current density: 0.54 mA/cm^2^, stirring speed: 150 rpm
**Supporting electrolyte type**	15 mM NaCl, 15 mM KCl, 10 mM Na_2_SO_4_	initial pH:4, initial arsenic concentration: 50 mg/L, current density: 0.54 mA/cm^2^, stirring speed: 150 rpm
**Stirring speed, rpm**	50, 150, 250, 350	initial pH:4, initial arsenic concentration: 50mg/L, current density: 0.54 mA/cm^2^

## Results and discussion

### Effect of initial pH

**Figure 2:**
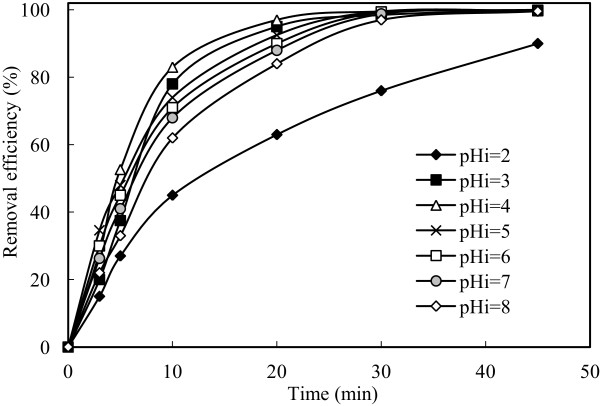
**Effect of pH on the arsenic removal efficiency (initial arsenic concentration: 50 mg/L, current density: 0.54 mA/cm**^**2 **^**and stirring speed: 150 rpm).**

**Figure 3:**
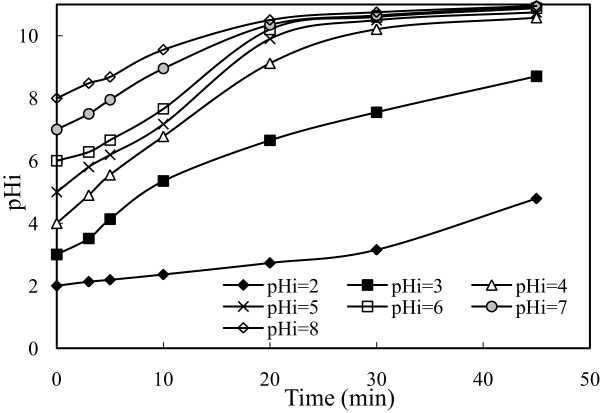
**Changes in system pH according to time at different initial pH (initial arsenic concentration: 50 mg/L, current density: 0.54 mA/cm**^**2**^**, stirring speed: 150 rpm, t = 20 min.).**

Initial pH is one of the important factors in affecting the performance of electrochemical process, increased during the study. To investigate this effect, a series of experiments performed under conditions of which values are given in Table 1. The results are presented in Figure 2. Initial pH of the solution affected the arsenic removal efficiency. At initial pH values with the range 2-8, current density of 0.54 mA/cm^2^, stirring speed of 150 rpm, arsenic removal efficiency was obtained as 63.00%, 95.00%, 97.00%, 92.50%, 89.50%, 88.00%, 84.00%, respectively at the end of 20 minutes. Under these conditions at initial pH values with the range 3-8, arsenic removal efficiency was reached above 99.00% at the end of 30 minutes. Even, at initial pH of 2 in which removal efficiency was lower than the others, arsenic removal efficiency was reached as 76% at the end of 30 minutes. At all initial pHs it was reached removal efficiency of 99.99% at the end of 60 minutes.Solubility of metal hydroxide species (both arsenic and iron hydroxides) strongly depends on the chemistry of the aqueous media. Removal of arsenic by electrocoagulation is significantly affected by solution pH. Both initial pH and the elevation of pH during electrocoagulation affect arsenic solubility and hence its removal. For better understanding of this situation, changes in pH of the solution during the process in the all experiments were observed and presented in Figure 3. As shown in Figure 3, the pH value increases as the time of electrocoagulation process is increased.

**Figure 4:**
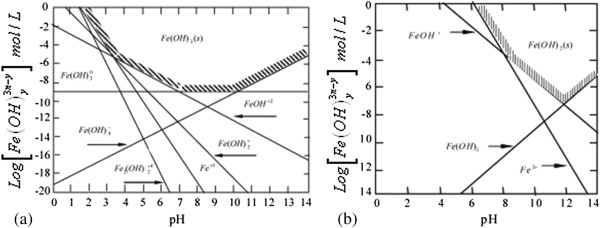
Aqueous-solid phase equilibrium for Fe(III) (a) and Fe(II) (b) species at infinite dilution.

**Figure 5:**
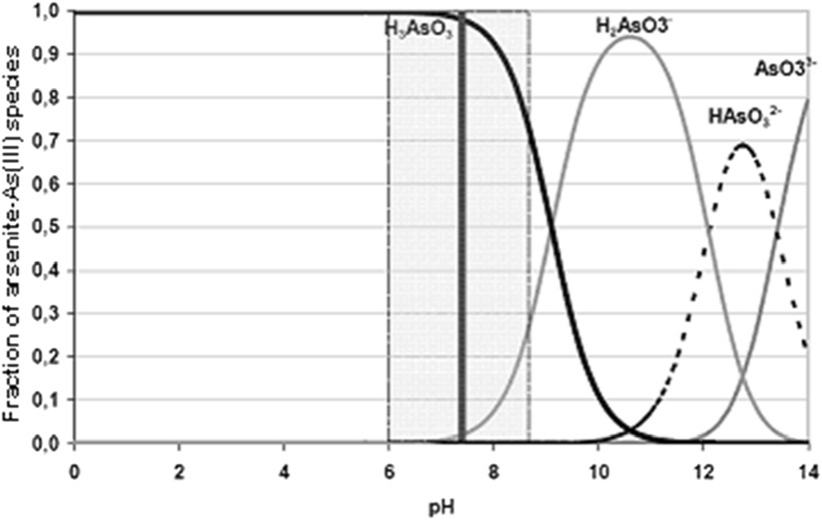
Distribution of arsenite species as a function of pH.

**Figure 6:**
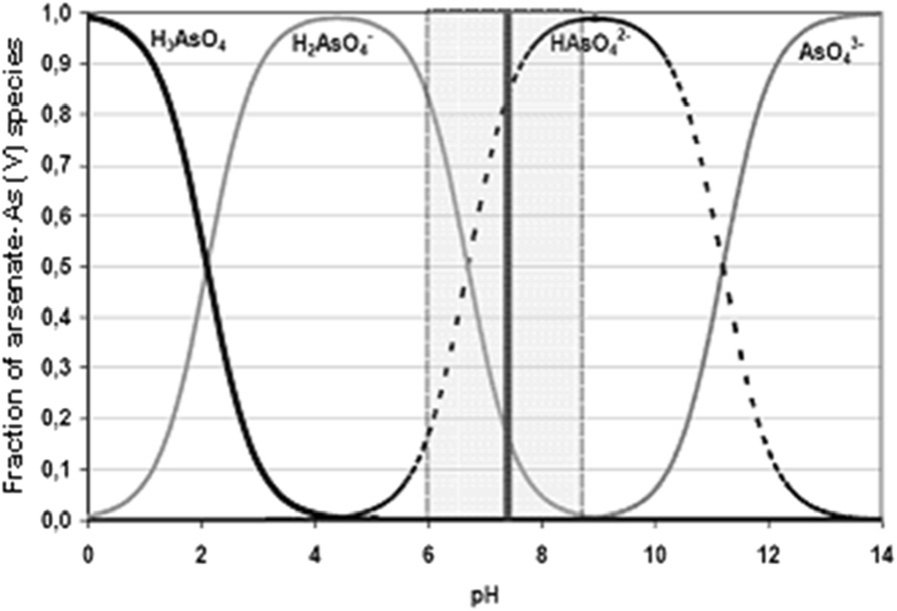
Distribution of arsenate species as a function of pH.

This happened because the OH^−^ ion accumulates in aqueous solution during the process. At the beginning pH increased rapidly and after a while it stopped when it reached to over 10.50 (initial pH of 4-8). Arsenic removal depends on both the initial and final pH of solution. Solubility diagram of iron (Fe(II), Fe(III)) according to pH and speciation of arsenite and arsenate (As(III) and As(V)) as a function of pH were given in Figures 4, 5 and 6, respectively [35,36]. As shown in Figure 4, the resolution of Fe(OH)_3_ is constant in all pH and its value is 10^−9^. Fe(OH)_2_ begins to form at approximately pH 5.

As a result of using iron electrodes in electrocoagulation, Fe^2+^ and Fe^3+^ ionswere produced by anodic dissolution and Fe(OH)_2_ and Fe(OH)_3_ flocks developed in the medium_._ Two mechanisms of the production of formed iron hydroxide were given in the 1-8 reactions [28]. It has been reported that in all the type of iron hydroxide, Fe(OH)_3_ may collapse and has effective in flock formation and adsorbe the pollutants. The best removal efficiency was obtained in pH of 8.0–8.5 in which Fe(OH)_3_ is the most stable. In this study, the reason of the significant increase of the removal of arsenic ions is due to the formation of the flocks of Fe(OH)_3(s)_during electrolysis.The chemistry of arsenic is quite complex and interesting, as it can be stable in four oxidation states, continue changing its states and its removal is dependent on pH of the medium, oxidation state and redox potential. In the aqueous environment, inorganic arsenic appears commonly in forms of arsenite (As(III)) and arsenate (As(V)). pH, redox potential and the presence of complexing ions such as ions of sulfur, iron, and calcium determine the arsenic valence and speciation. Figures 5 and 6 contain a summary of the forms of arsenic typically present in water.

In typical drinking water pH ranges of 6 to 9, the predominant arsenite species in neutral in charge (H_3_AsO_3_) while arsenate species are present as (H_2_AsO_4_^−^ and HAsO_4_^2−^). In oxygenated waters, As(V) is dominant, existing in an ionic forms either H_2_AsO_4_^−^ or HAsO_4_^2−^over the pH range typically encountered in water treatment. Under anoxic conditions, As(III) is stable with nonionic (H_3_AsO_3_) and anionic (H_2_AsO_3_^−^) species dominant below and above pH 9.2 [37]. Due to the differences in ionic charge of the arsenite and arsenate particles in the pH 6 to 9 range the neutrally charged arsenite compound (H_3_AsO_3_) is difficult to remove when compared to the divalent (HAsO_4_^2−^) and monovalent arsenate anions (H_2_AsO_4_^−^). The negative charges of the arsenite and arsenate compounds make arsenic easy to remove by adsorptive, co-precipitate and chemical exchange processes. As(V) species are negatively charged above pH 2.1, whereas negatively charged As(III) species do not predominate until pH levels exceed 9.2.

In this study, at initial pH of 2, final pH reached to 3.15 at the end of 30 minutes during the process. At this pH arsenic was found as nonionic arsenite (H_3_AsO_3_) or arsenate anions (H_2_AsO_4_^−^). Arsenic removal efficiency was found as 97.91% at thirtieth minutes so its molecular charge must be negative and arsenic was as H_2_AsO_4_^−^. At initial pH of 3, final pH reached to 7.89 at the end of 30 minutes during the process. At this pH arsenic was found as nonionic arsenite (H_3_AsO_3_) or arsenate anions (H_2_AsO_4_^−^ and HAsO_4_^2−^). Arsenic removal efficiency was found as 98.42% at thirtieth minutes so its molecular charge must be negative and arsenic was as H_2_AsO_4_^−^ and HAsO_4_^2−^. At initial pH of 4, 5, 6, final pH reached to minimum 10.51 and maximum 10.70 at the end of 30 minutes during the process. In the range of pH 4.00 to 10.70 arsenic was found as arsenite (H_3_AsO_3_, H_2_AsO_3_^−^) or arsenate anions (H_2_AsO_4_^−^, HAsO_4_^2−^). Arsenic removal efficiency was found as over 99% at thirtieth minutes so its molecular charge must be negative and arsenic was as H_2_AsO_3_^−^, H_2_AsO_4_^−^, HAsO_4_^2−^. At initial pH of 7 and 8 final pH reached to 10.74 ve 10.75 respectively at the end of 30 minutes during the process. In the range of pH 7.00 to 10.75 arsenic was found as arsenite (H_3_AsO_3_, H_2_AsO_3_^−^) or arsenate anions (HAsO_4_^2−^). Arsenic removal efficiency was found respectively as 98.78% and 98.00% at thirtieth minutes so its molecular charge must be negative and arsenic was as H_2_AsO_3_^−^, HAsO_4_^2−^. It is known that ferric hydroxides have a higher adsorption capacity for As(V) than for As(III) when the water pH is lower than about 8 [38]. Based on this, As(III) might be oxidized to As(V) and arsenic might be as HAsO_4_^2−^. Because of its molecular charge was negative, it pulled the positively charged metal hydroxides electrostatically and arsenic was removed from solution with great efficiency easily [39]. In this electrocoagulation process the removal mechanism of arsenic was: oxidation of As(III) to As(V) and subsequent removal by adsorption/co-precipitation with iron (III) hydroxide generated in the process. The reaction of arsenate co-precipitated with or adsorbed by the (FeOH) colloids can be written as [40]:

(13) $$2\mathrm{FeOOH}+H_{2}\mathrm{As}{O_{4}}^{-}\to {\left(\mathrm{FeO}\right)}_{2}\mathrm{HAs}O_{4}+H_{2}O+OH^{-}$$

**Figure 7:**
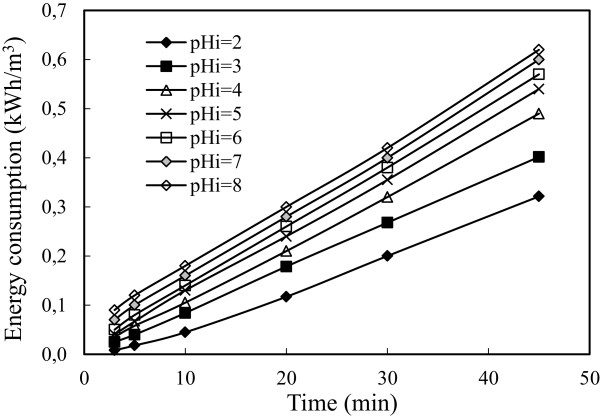
**Effect of pH on the energy consumption (initial arsenic concentration: 50 mg/L, current density: 0.54 mA/cm**^**2 **^**and stirring speed: 150 rpm).**

Electrical energy consumption values were calculated from Eq. (12) and the relationship between the energy consumption and pH is shown in Figure 7. The lowest energy consumption curve was obtained in the experiments carried out with initial pH of 2 because the solution had the highest conductivities. Electrical conductivity caused to decrease energy consumption [41]. The pH of As_2_O_3_ solution containing 50 mg/L arsenic was about 11.34 and its conductivity was about 460 μS/cm. The decreasing pH of solution by adding H_2_SO_4_ caused to rise of electrical conductivity. Thus, high conductivity values of solution caused to low resistance values and low energy consumption. Also in the initial pH value of 4, at the end of 30 minutes it was obtained the highest arsenic removal and low energy consumption (0.33 kWh/m^3^) so the optimum initial pH was selected as 4.

### Effect of current density

**Figure 8:**
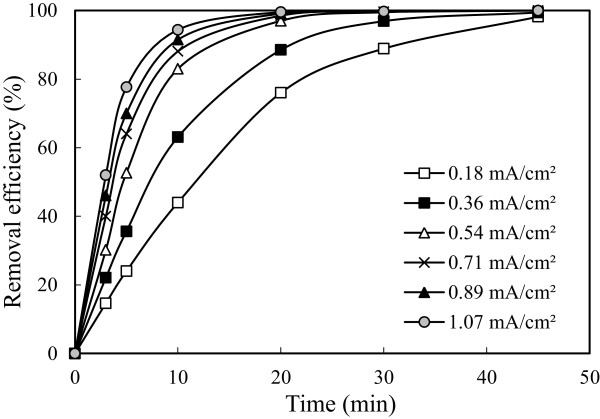
Effect of current density on the arsenic removal efficiency (initial arsenic concentration: 50 mg/L, initial pH: 4 and stirring speed: 150 rpm).

The current density is defined as the ratio of current input to the electrolytic cell from the surface area of the electrode. In all electrochemical processes, current density is the most important parameter for controlling the reaction rate within the reactor. It is well known that the amount of current density not only determines the coagulant dosage rate but also the bubble production rate, size and the flocks’ growth, which can influence the treatment efficiency of the electrocoagulation [42]. To investigate the effect of current density, a series of experiments performed under conditions in which are given in Table 1. The results are presented in Figure 8.

Increasing the current density from 0.18 mA/cm^2^ to 1.07 mA/cm^2^ the arsenic removal efficiency is further improved. The dissolution rate of iron increased with current density increased and thus fixed amount of pollutants reacted to more Fe(OH)_3_ and so more pollutants were removed. At the higher current density, especially 0.89 and 1.07 mA/cm^2^, lower removal efficiency was obtained than expected. The reason of this matter was thought that, in experiments high current density applied, on account of the amount of sludge consisted in reactor was too much and enough iron didn’t resolution because of the excessive amount of sludge in the minutes following the time is considered.

**Figure 9:**
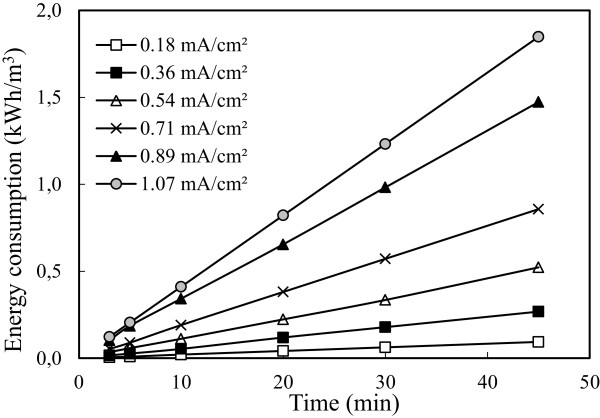
Effect of current density on the energy consumption (initial arsenic concentration: 50 mg/L, initial pH: 4 and stirring speed: 150 rpm).

Although that current density was increased from 0.18 mA/cm^2^ to 1.07 mA/cm^2^ increased from 88.88% to 99.68% of arsenic removal efficiency, energy consumption reached from 0.06 to 1.23 kWh/m^3^ at the end of 30 minutes. The obtained results for arsenic removal were demonstrated in Figures 8 and 9. High electrical energy consumption with increasing current density was an expected result because energy consumption impressed linearly current density as seen in Eq. 12. Although higher current density caused to solve more electrode material and remove more pollutant, this state was not desired for electrical energy consumption. As shown in Figure 8, same removal efficiency was obtained in the current density of 0.54 mA/cm^2^ to 1.07 mA/cm^2^ at the end of 30 minutes duration. Since the values were very close to each other, working at current density of 0.54 mA/cm^2^ was more appropriate in terms of cost, so current density of 0.54 mA/cm^2^ was preferred in experiments.

### Effect of initial arsenic concentration

**Figure 10:**
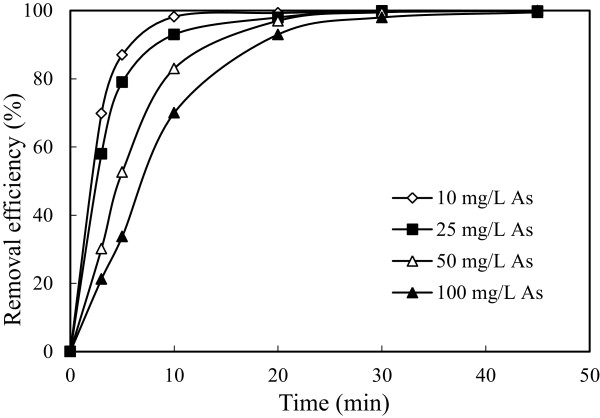
**Effect of initial arsenic concentration on the arsenic removal efficiency (initial pH: 4, current density: 0.54 mA/cm**^**2 **^**and stirring speed: 150 rpm).**

**Figure 11:**
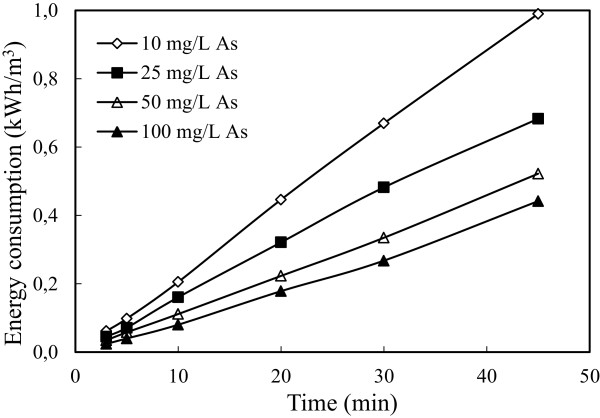
**Effect of initial arsenic concentration on the energy consumption (initial pH: 4, current density: 0.54 mA/cm**^**2 **^**and stirring speed: 150 rpm).**

To investigate effect of initial arsenic concentration, a series of experiments performed under conditions in which are given in Table 1. The results are presented in Figure 10. The obtained experimental data showed that increasing initial arsenic concentration decreased arsenic removal efficiency. This can be explained as following; although the same amount Fe^3+^ passed to solution at the same current density for all arsenic concentration, Fe^3+^ was insufficient for solutions including higher arsenic concentration. The solution conductivity increased with increasing arsenic concentration. As a result of this situation, applied potential and energy consumption decreased. The results obtained were shown graphically in Figure 11.

### Effect of supporting electrolyte type

**Figure 12:**
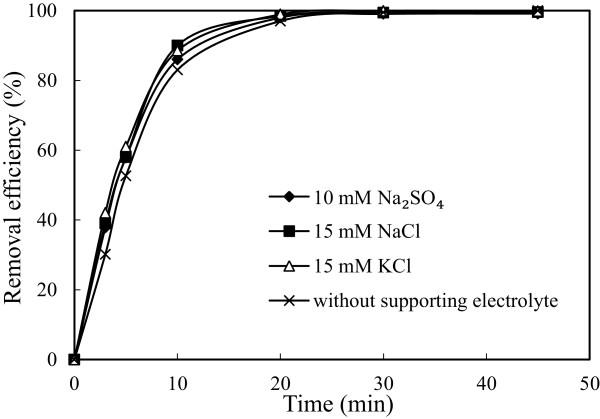
**Effect of supporting electrolyte type on the arsenic removal efficiency (initial arsenic concentration: 50 mg/L, initial pH: 4, current density: 0.54 mA/cm**^**2 **^**and stirring speed: 150 rpm).**

**Figure 13:**
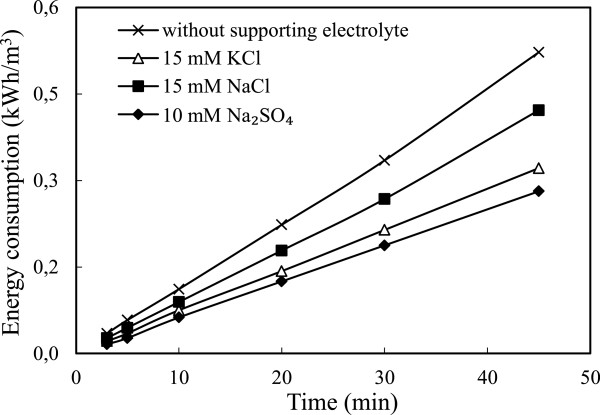
**Effect of supporting electrolyte type on the energy consumption (initial arsenic concentration: 50 mg/L, initial pH: 4, current density: 0.54 mA/cm**^**2 **^**and stirring speed: 150 rpm).**

To investigate this effect, a series of experiments were performed under conditions in which are given in Table 1. The results are presented in Figure 12. Adding supporting electrolyte had not significant effects on the arsenic removal. Supporting electrolyte decreased energy consumption because amounts of ions in solution increased, applied potential decreased and the conductivity of solution increased under constant current density. Electrical energy consumption values were calculated from Eq.12 and the data are shown in Figure 13. The obtained results showed that the most favorable supporting electrolyte type was Na_2_SO_4_ for arsenic removal obtained lowest with Na_2_SO_4_.

### Effect of stirring speed

**Figure 14:**
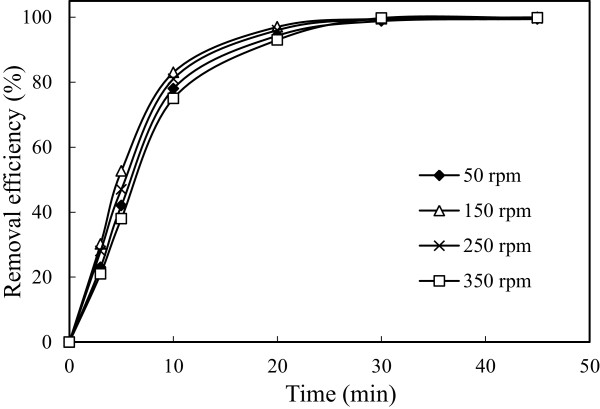
**Effect of stirring speed on the arsenic removal efficiency (initial arsenic concentration: 50 mg/L, initial pH: 4, current density: 0.54 mA/cm**^**2**^**).**

**Figure 15:**
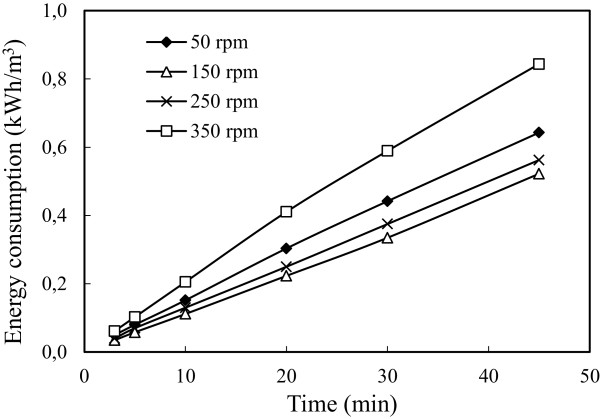
**Effect of stirring speed on the energy consumption (initial arsenic concentration: 50 mg/L, initial pH: 4, current density: 0.54 mA/cm**^**2**^**.**

To investigate stirring speed effect, a series of experiments performed under conditions in which are given in Table 1. The results are presented in Figure 14. Increasing stirring speed decreased arsenic removal efficiency because increasing stirring speed decreased capability of flock formation of iron ions. The stirring speed, smaller than 150 rpm, decreased arsenic removal efficiency and this speed did not supply a homogeneous mixture in the reactor. The energy consumption values were calculated and were shown in Figure 15. The energy consumption values increased contrary to the arsenic removal efficiency both for the stirring speed above 150 rpm (250, 350 rpm) and below 150 rpm (50 rpm). Graphical results showed that the flocks deposited between electrodes because the flocks couldn’t mix homogeneously and this deposition caused to the increment of cell resistance at low stirring speed.

The increase in the cell resistance causes the increase of potential value in the systems where constant current density and this causes the increase of the amount of energy consumption per unit volume. It was considered that, the reason of more energy consumption due to the higher stirring speed, is a result of high speed consisted in the reactor created negative pressure on the flow of electrons which slows the flow of electrons or creates an additional resistance. The stirring speed of 250 and 350 rpm are good in terms of efficiency but they were not preferred in terms of energy consumption. So the best stirring speed was 150 rpm for arsenic removal.

## Conclusions

The present study clearly demonstrated the applicability of electrocoagulation process using the iron electrode for arsenic removal. The effects of operational parameters such as initial pH, electrolysis time, current density, initial arsenic concentration, supporting electrolyte type and stirring speed on arsenic removal efficiency were studied in detail and explained as well. It has been observed that the pH is an important operating factor influencing the performance of electrocoagulation process. Optimal initial pH was found as 4 in the use of iron as sacrificial electrode material in the treatment. Arsenic was as arsenate anions (HAsO_4_^2−^) and their molecular charge were negative; they pulled the positively charged metal hydroxides electrostatically so arsenic was removed from solution with great efficiency easily. The removal mechanism of arsenic was oxidation of As(III) to As(V) and subsequent removal by adsorption/co-precipitation with iron (III) hydroxide generated in the process. Increasing the current density 0.18 mA/cm^2^ to 1.07 0 mA/cm^2^, arsenic removal efficiency increased from 88.88% to 99.68%, energy consumption reached from 0.06 to 1.23 kWh/m^3^. Increasing current density increased amount of Fe^3+^ ions and Fe^3+^ ions reacted with more arsenic (arsenite or arsenate) ions in aqueous media. Arsenic removal efficiency decreased with increasing arsenic concentration. Increasing arsenic concentration increased conductivity of solution. The higher conductivity values decreased energy consumption. Adding supporting electrolyte had not significant effects on the arsenic removal. Supporting electrolyte decreased energy consumption because amounts of ions in solution increased, applied potential decreased and the conductivity of solution increased under constant current density. Stirring speed affected arsenic removal efficiency. Arsenic removal decreased when both stirring speeds fewer than 150 rpm were not proved homogenization in the reactor. The best stirring speed was 150 rpm for arsenic removal. Stirring speed above 150 rpm prevented formation of Fe(OH)_3_ and to react between arsenic and Fe^3+^ ions species.

As a result under the optimum conditions of an initial pH of 4, current density of 0.54 mA/cm^2^, stirring speed of 150 rpm, electrolysis time of 30 minutes, arsenic removal was obtained as 99.50. Electrical energy consumption in the above conditions was calculated as 0.33 kWh/m^3^. Electrocoagulation with iron electrodes was able to bring down 50 mg/L arsenic concentration to less than 10 μg/L at the end of electrolysis time of 45 minutes with low electrical energy consumption as 0.52 kWh/m^3^. It can be concluded from this study that electrocoagulation with iron electrodes is a promising technique for arsenic removal.

## Competing interests

The authors declare that they have no competing interests.

## Authors’ contributions

BZC carried out the experiments under the guidance of RB, AEY and BAF. BZC, RB, AEY and BAF compiled the experimental data in journal format. All authors read and approved the final manuscript.
